# Aromaticity in Ancient Zeise’s Salt

**DOI:** 10.1021/acs.inorgchem.5c03091

**Published:** 2025-09-29

**Authors:** Ankur K. Guha, Amlan J. Kalita, Mesías Orozco-Ic, María A. Fernández-Herrera, Gabriel Merino

**Affiliations:** † Advanced Computational Chemistry Centre, Department of Chemistry, Cotton University, Panbazar, Guwahati, Assam 781001, India; ‡ Department of Chemistry, Nabajyoti College, Kalgachia, Assam 781319, India; § Instituto de Ciencias Físicas, Universidad Nacional Autónoma de México, Cuernavaca 62210, México; ∥ Departamento de FísicaAplicada, Centro de Investigación y de Estudios Avanzados, 130299 Unidad Mérida, Km 6 Antigua Carretera a Progreso. Apdo. Postal 73, Cordemex, Mérida, Yucatán 97310, México

## Abstract

Zeise’s salt, K­[(C_2_H_4_)­PtCl_3_]·H_2_O, is historically recognized as the first synthesized organometallic compound. Its bonding is commonly described by the Dewar–Chatt–Duncanson model. We analyzed the [(C_2_H_4_)­PtCl_3_]^−^ anion using magnetic response calculations, molecular orbital decomposition, and energetic criteria. Out-of-plane orbitals contribute minimally, while several in-plane orbitals generate strong diatropic currents, indicating a dominant σ-aromatic character. Isodesmic reaction calculations show that the formation of the C–Pt–C ring is energetically favorable, further supporting delocalization beyond ring strain. Electron delocalization assessed with the EDDB_F_ method indicates 1.8 |e| delocalized over the C–Pt–C fragment. These results support considering Zeise’s salt not only as the first organometallic compound but also as a σ-aromatic metallacycle.

## Graphical abstract

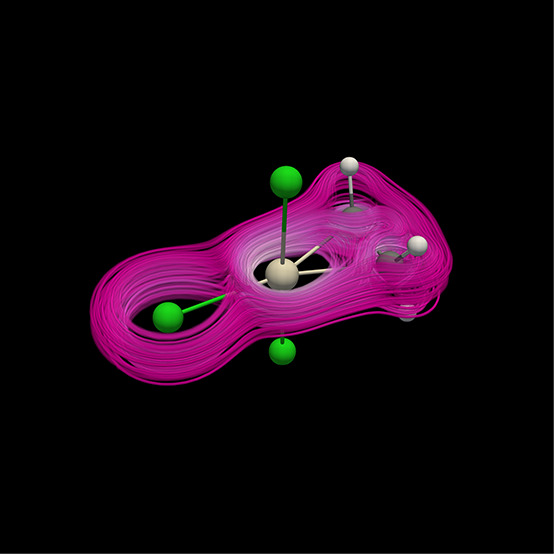

## Introduction

Potassium trichloro­(ethylene)­platinate­(II), K­[(C_2_H_4_)­PtCl_3_]·H_2_O, is historically recognized as the first synthesized organometallic compound (ca. 1820).^1,2^ Despite its early discovery, the bonding and molecular structure remained unclear for over a century, fueling intense debate.^3,4^ A turning point occurred in the 1950s with the Dewar–Chatt–Duncanson (DCD) model,^5,6^ which describes the Pt-ethylene interaction in the [(C_2_H_4_)­PtCl_3_]^−^ anion as a synergistic mechanism. According to this model, electron density is donated from the filled in-plane π-orbital of ethylene to an empty Pt *d*-orbital, forming a σ-bond, while back-donation occurs from a filled Pt *d*-orbital into the ethylene π*-orbital. This electron redistribution elongates the C–C bond by approximately 4% and shifts its stretching frequency to lower values. Optimal *d*-π* overlap requires the ethylene moiety to adopt an orientation perpendicular to the PtCl_3_ plane. X-ray and neutron diffraction studies, nearly 150 years after the synthesis, confirmed both the DCD model and the structure of Zeise’s salt.^7,8^ Vibrational spectroscopy,^9^ ultraviolet absorption spectroscopy in solution,^10–12^ and gas-phase photoelectron spectroscopy^13^ provide additional support, while theoretical studies have refined the understanding of its electronic structure, establishing Zeise’s salt as a prototypical transition metal-olefin system.^14–17^

Let us consider an intriguing (albeit ultimately flawed) proposal. The PtCl_3_^–^ fragment is isolobal with singlet CH_2_, implying that the resulting three-membered metallocycle could electronically resemble cyclopropane. However, the orbital types (*d* vs *sp* ^3^) and bonding interactions (dative-covalent vs purely covalent) differ. Aromaticity has been proposed as a stabilizing factor in cyclopropane^18–22^ and various organometallic systems,^23–27^ particularly three-membered metallacycles.^28–34^ This combination of the “isolobal” analogy between PtCl_3_^–^ and CH_2_, potential σ-aromatic character, and identification of aromaticity in organometallic rings raises the question of whether Zeise’s salt could be described as a σ-aromatic metallacycle.

## Results and Discussion

To ensure a reliable structural description before analyzing bonding and aromaticity, eight functionals were benchmarked using the def2-QZVP basis set (see Computational Details).^35–43^ All yielded similar geometries in close agreement with experimental data,^7,8^ indicating consistent descriptions. Although M06-2X^43^ showed slightly larger deviations (maximum bond distance error of 0.03 Å), it was the only functional to reproduce a key feature: the longer *trans* Pt–Cl bond relative to the *cis* Pt–Cl bonds (Figure 1a). The C–C bond elongation (1.396 vs 1.322 Å in free ethylene) and the *trans* Pt–Cl bond lengthening are consistent with this *trans* effect.

**Figure 1:**
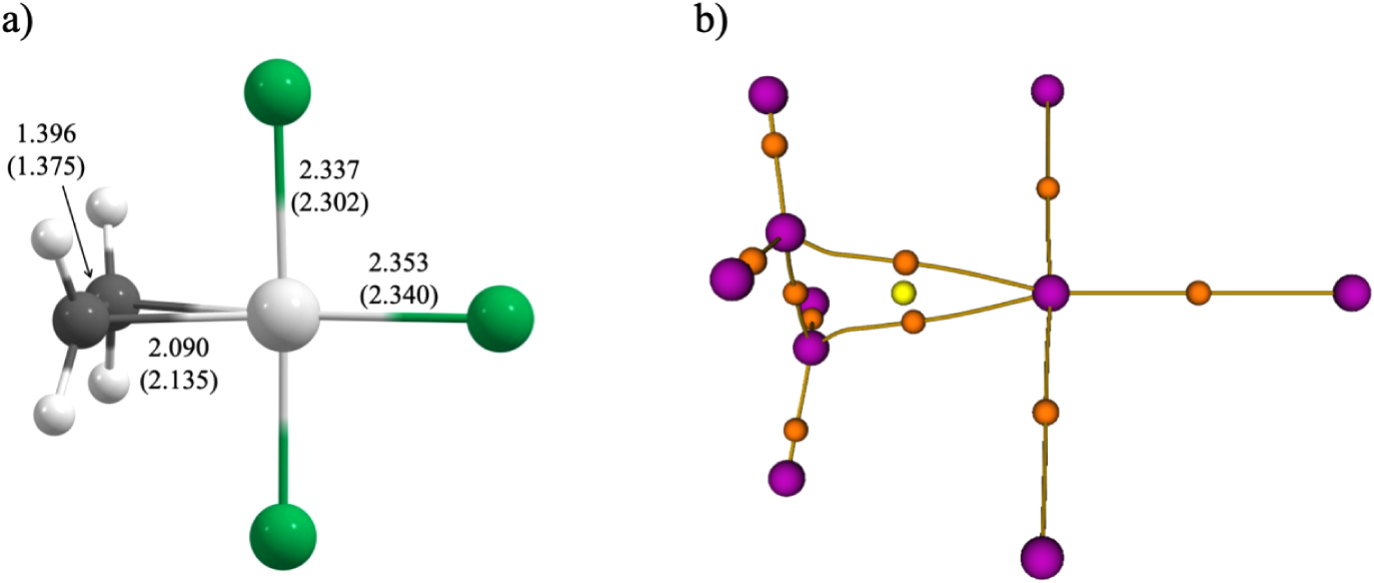
(a) M06-2X/def2-QZVP geometry of the [(C_2_H_4_)­PtCl_3_]^−^ anion. Bond lengths are given in Å, with experimental values shown in parentheses for comparison. Carbon is shown in gray, hydrogen in white, chlorine in green, and platinum in silver. (b) Molecular graph of the [(C_2_H_4_)­PtCl_3_]^−^ anion. Attractors are shown in pink, critical points (3,–1) in orange, and the critical point (3,+1) in yellow.

With the methodology established, the next question was whether Zeise’s salt should be described as a metallocyclopropane or as an olefin π-complex. Interatomic distances alone were inconclusive, and carbon pyramidalization provided limited information. The dissociation of this salt into PtCl_3_^–^ and ethylene requires 36.9 kcal/mol (including the zero-point correction) and does not allow determination of whether there is a ring or not. Because aromaticity requires a cyclic framework,^44^ the first step was to verify the existence of a three-membered C–Pt–C ring. Electron density analysis^45^ revealed two gradient paths connecting Pt to the carbon atoms and a ring critical point, consistent with the Poincaré–Hopf relationship (Figure 1b). The descriptors at the Pt–C (3,–1) critical points (ρ_Pt–C_ = 0.12 au, ∇^2^ρ_Pt–C_ = 0.14 au) classify these contacts as closed-shell interactions. ρ at these critical points is about half that at the C–C (3,–1) critical points. As shown in Figure 1b, the gradient paths connecting Pt to the carbon atoms display pronounced curvature, and the corresponding critical points exhibit high ellipticity (ϵ=0.39) . These features suggest the presence of multicenter bonding. Together, they support η^2^-coordination through two C–Pt bond paths and confirm the existence of a cyclic C–Pt–C structure (Figure 1b).

Energetic and magnetic criteria were then considered to assess the aromatic stabilization. Isodesmic reactions were used to quantify the stabilization energy. While cyclopropane shows σ-aromatic character, ring strain dominates,^46^ as reflected by a highly exothermic conversion to pentane (Δ*E* = −24.4 kcal/mol, computed at the M06-2X/def2-QZVP level).^43^ In contrast, analogous bond-cleavage reactions for Zeise’s salt (Scheme 1) are endothermic (+12.8 kcal/mol for Pt–C and +11.5 kcal/mol for C–C, computed at the M06-2X/def2-QZVP level), indicating that electron delocalization stabilizes the C–Pt–C ring beyond its inherent strain.

**Scheme 1:**
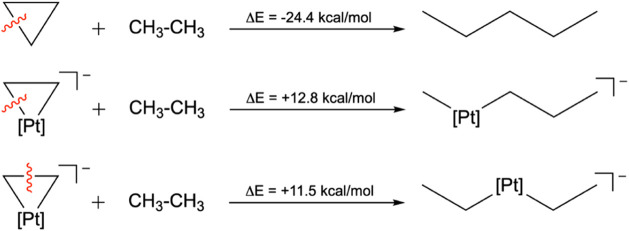
Isodesmic Reactions Used to Evaluate Energy Stabilization (Computed at the M06-2X/def2-QZVP Level)^a^

a [Pt] denotes the fragment PtCl_3_^–^.

Magnetic criteria provide complementary evidence. In cyclopropane, σ-electrons generate a diatropic ring current outside the ring, a paratropic current inside, and localized vortices around carbon atoms due to the bent σ-bonds. However, this σ-aromaticity is largely offset by ring strain, and previous studies have found no clear σ-aromatic stabilization relative to acyclic references,^22^ while the intensity of its σ-ring current is comparable to that in π-aromatic rings.^21^

Let us now examine the magnetic response of Zeise’s salt. First, it is necessary to define the molecular orientation to avoid confusion. The C–Pt–C triangle defines the plane of interest, which is usually assigned as the *xy* plane, with an external field (**B** ^ext^) applied along the *z*-axis. Because the system has *C*_2*v*_ symmetry, with the principal axis passing through Pt and bisecting the C–C bond, the *z*-axis was aligned with the principal axis. Consequently, the C–Pt–C triangle lies in the *xz* plane, and **B** ^ext^ was applied along the *y* direction, perpendicular to the molecular plane.

With this orientation defined, the magnetically induced current density^47–49^ (**J** ^ind^) and the induced magnetic field (**B** ^ind^)^50–52^ were computed for the [(C_2_H_4_)­PtCl_3_]^−^ anion with **B** ^ext^ perpendicular to the C–Pt–C plane. **J** ^ind^ circulates mainly outside the triangle (Figure 2a), and the *y*-component of **B** ^ind^ (*B* ^*ind*^_*y*_) shows strong shielding (below −50 ppm) within and above the ring (Figure 2b). Applying the removing valence electron (RVE) approximation^53–55^ revealed that core electrons contribute only localized shielding near Pt (Figure 2b). The computed ring-current strength (13.94 nA/T, core electrons removed) is entirely diatropic and exceeds that of cyclopropane (10.19 nA/T). Furthermore, unlike cyclopropane, the C–Pt–C ring does not experience significant ring strain, supporting its aromatic stabilization.

**Figure 2:**
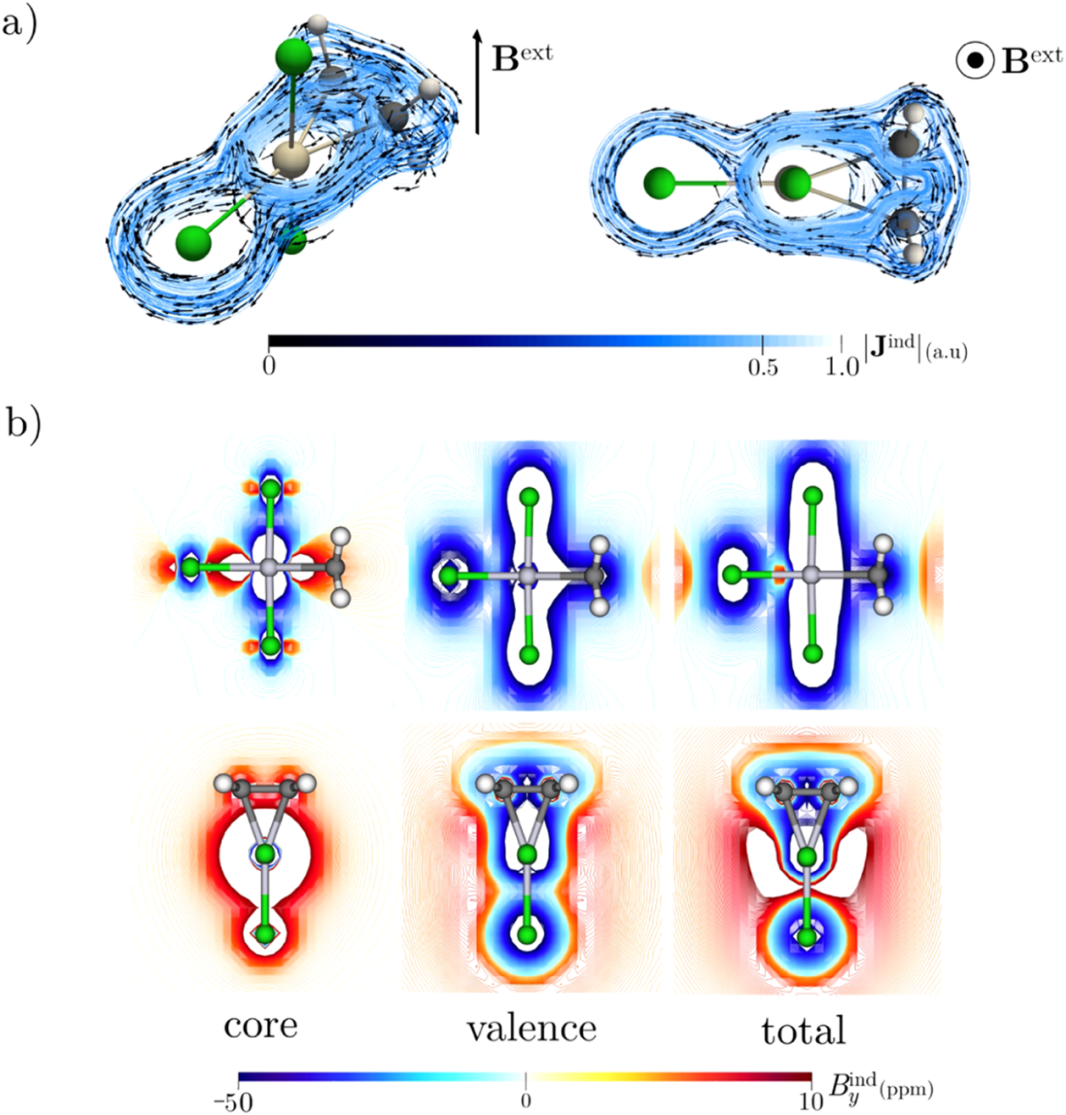
(a) Magnetically induced current density **J** ^ind^ maps for the [(C_2_H_4_)­PtCl_3_]^−^ anion. Arrows indicate the direction of the current density. Atom colors are the same as those in Figure 1. The |**J** ^ind^| scale is in atomic units (1 au = 100.63 nA/T Å^2^); (b) *B* ^*ind*^_*y*_ isolines plotted in the molecular plane (bottom) and a transverse plane (top) of the [(C_2_H_4_)­PtCl_3_]^−^ anion. The external magnetic field is oriented perpendicular to the C–Pt–C ring plane, as shown in the plots in (a).

One of the central questions is whether this system should be described as a metallacyclopropane or an olefin π-complex. This issue is closely related to the origin of its magnetic response, which suggests an aromatic character. Because the response can be decomposed into orbital contributions, both questions can be addressed through molecular orbital analysis. Figure 3a shows the 22 valence orbitals of the [(C_2_H_4_)­PtCl_3_]^−^ anion, with their corresponding irreducible representations. According to the DCD model, bonding involves σ donation from the ethylene π orbital to 2a_1_ of Pt and π back-donation from the Pt b_2_ orbital into the ethylene π* orbital (Figure S1). The contribution of each orbital was evaluated along a *B* ^*ind*^_*y*_ profile perpendicular to the C–Pt–C plane, starting from the geometric center of the triangle, defined as the average position of the three atoms (see Figure 3b).

**Figure 3:**
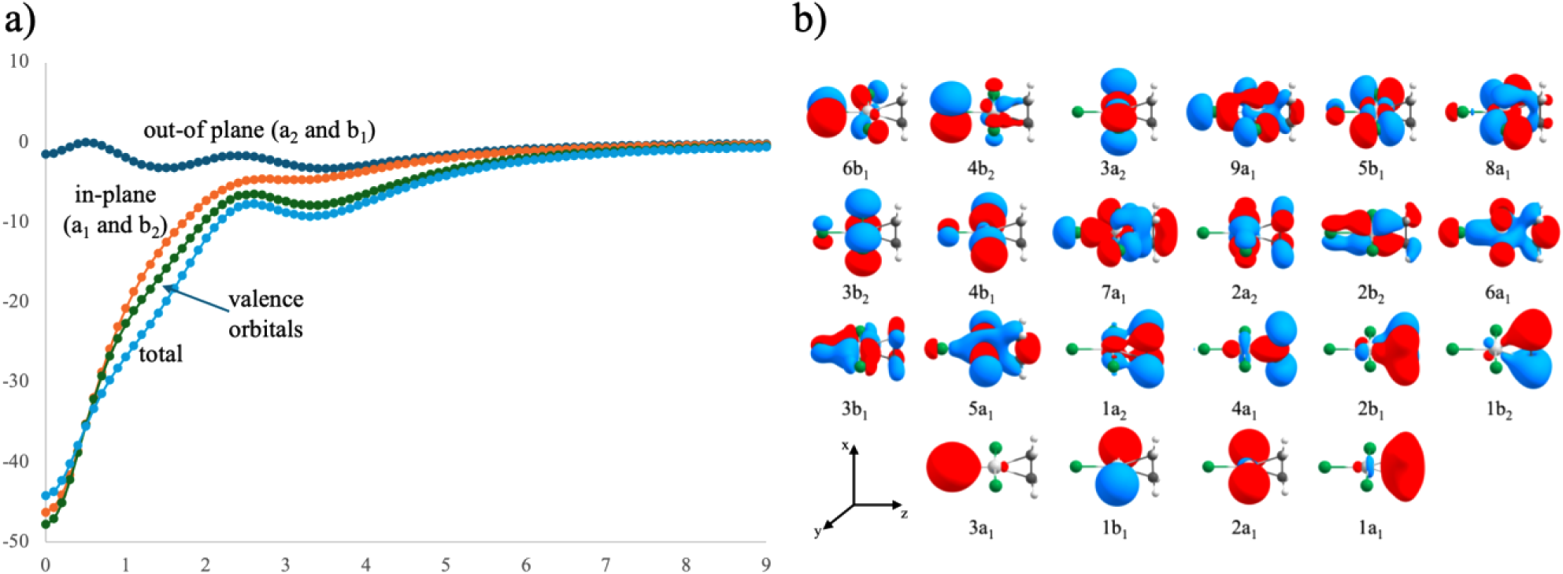
(a) Valence orbitals of the [(C_2_H_4_)­PtCl_3_]^−^ anion with irreducible representations. (b) *B* ^*ind*^_*y*_ profile perpendicular to the C–Pt–C triangle.

If molecular orbitals with a_2_ or b_1_ symmetry contributed to the magnetic response, their effects would appear out of the C–Pt–C plane. In practice, only three of the eight out-of-plane orbitals show a notable response: 2b_1_ (−14.9 ppm, diatropic), 1a_2_ (4.6 ppm, paratropic), and 2a_2_ (10.5 ppm, paratropic). The paratropic contributions largely cancel the diatropic effect of 2b_1_. Other out-of-plane orbitals produce negligible responses, resulting in a weak net diatropic out-of-plane response (−1.55 ppm at the ring center, decreasing slightly to −3.2 ppm at 1.5 Å above or below the molecular plane).

In the C–Pt–C plane, the orbitals involved in π back-donation are 4b_2_ and 2b_2_. The response from 4b_2_ is small (2.9 ppm), while 2b_2_ produces a stronger diatropic response (−12.0 ppm). This difference arises from the relative phase of the *trans* Cl *p* orbital; when it is in phase, the response becomes more intense. The orbitals associated with σ donation from the filled alkene π orbital to Pt are 9a_1_, 8a_1_, and 7a_1_, which include contributions from Cl *p* orbitals that modulate overlap with the π* orbital. The 9a_1_ and 8a_1_ produce minor responses (3.6 and −3.8 ppm), likely due to steric repulsion from the *cis* Cl atoms. In contrast, 7a_1_ shows a stronger diatropic response (−11.5 ppm), consistent with the effective overlap between the Cl *p* orbitals and the ethylene π cloud. Orbitals localized mainly on the PtCl_3_^–^ fragment or the ethylene moiety contribute minimally. The largest contribution arises from 1a_1_ (−27.6 ppm), while the remaining a_1_ and b_2_ orbitals contribute diatropically with lower intensity (around −18.7 ppm). These results show that the dominant magnetic response originates from in-plane orbitals, consistent with the σ-aromaticity and energetic data.

As noted by a reviewer, multicenter orbitals are single-electron wave functions that can be transformed into localized bonding representations if orthogonalized, and multiple orbitals may contribute to delocalization. Bonding or antibonding character is determined not by individual orbitals, but by their coherent interference. To address this, Szczepanik and coworkers proposed the electron density of delocalized bond (EDDB) method.^62^ In the present study, the EDDB_F_ variant was used to quantify the σ-bonding coherence. The EDDB_F_ contour for the C–Pt–C fragment at an isovalue of 0.01 |e| indicates a delocalized electron population of 1.8 |e|, corresponding to 0.6 |e| per atom (Figure 4). These results provide independent confirmation of the σ-aromatic character inferred from orbital and magnetic analyses, reinforcing the interpretation of Zeise’s salt as a σ-aromatic metallocycle.

**Figure 4:**
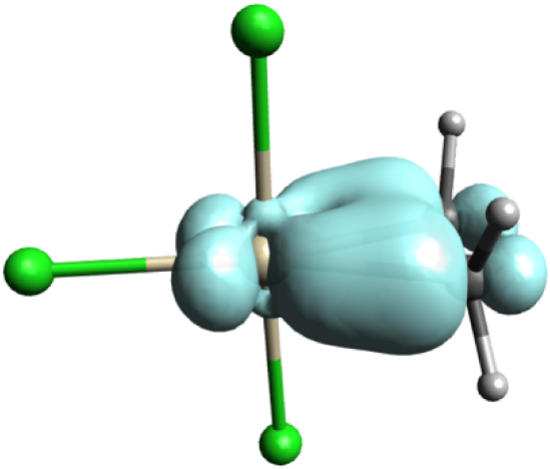
EDDB_F_ isosurfaces of the [(C_2_H_4_)­PtCl_3_]^−^ anion with an isovalue of 0.01 |e|.

## Conclusions

In summary, we have reexamined the bonding and electronic structure of Zeise’s salt, K­[(C_2_H_4_)­PtCl_3_]·H_2_O, combining structural, energetic, and magnetic analyses with molecular orbital and electron delocalization studies. The C–Pt–C fragment forms a well-defined three-membered ring, confirmed by the analysis of the electron density, which provides the cyclic framework required for potential aromaticity. Energetic analysis using isodesmic reactions indicates that the C–Pt–C ring is stabilized by delocalization, with bond cleavage being endothermic despite inherent ring strain.

Magnetic response calculations reveal that out-of-plane orbitals contribute minimally, whereas several in-plane orbitals generate strong diatropic currents, demonstrating dominant σ-aromatic character. Orbital decomposition along profiles perpendicular to the C–Pt–C plane shows that the magnetic response is largely driven by in-plane orbitals. Complementary EDDB_F_ analysis confirms 1.8 |e| delocalized over the three-membered ring, quantitatively supporting σ-aromaticity.

Overall, these results establish Zeise’s salt as a prototypical σ-aromatic metallocycle, where electron delocalization accounts for its unique stability. This study establishes that the first organometallic compound also represents an example of a σ-aromatic metallocycle and provides a framework for evaluating σ-aromaticity in other transition metal-olefin systems.

## Computational Details

We performed a benchmark to identify the functional that best reproduces the molecular structure for which experimental data were available. All calculations were performed by using the def2-QZVP basis set. The mean absolute errors (MAEs, in Å) for the eight functionals tested were BHandHLYP^35,36^ (0.0150), CAM-B3LYP^37^ (0.0168), ωB97XD^38^ (0.0170), TPSS^39^ (0.0200), MN15 ^40^ (0.0206), mPW1PW91 ^41^ (0.0206), PBE0 ^42^ (0.0214), and M06-2X^43^ (0.0252). Overall, the deviations were relatively small, indicating that all functionals provided a reasonable description of the structure. However, only M06-2X^43^ reproduced a key experimental feature: the *trans* Pt–Cl bond is longer than the *cis* Pt–Cl bonds. All other functionals predicted the opposite trend, despite their lower MAEs. Because correctly capturing this *cis*/*trans* relationship was essential for our analysis, we selected M06-2X for the study. Harmonic frequency analysis confirmed that the optimized structure corresponds to a true local minimum. We evaluated the energies of the isodesmic reactions at the M06-2X/def2-QZVP level, obtaining values of 12.8 and 11.5 kcal/mol. Electron density properties were examined within the framework of Bader theory.^45^ These results are close to those from ωB97XD with the same basis set (14.0 and 13.2 kcal/mol), differing by approximately 1.5 kcal/mol. All computations were carried out using Gaussian 16.^56^ Electron density using Bader theory was analyzed using the Multiwfn program code.^57^

Magnetically induced current density and induced magnetic field were computed at the BHandHLYP^35,36^ /x2c-TZVPall-2c^58^ level using the GIMIC^47–49^ and aromagnetic programs,^59^ respectively. Orbital contributions to **B** ^ind^ were obtained through the natural chemical shielding (NCS) analysis^60^ as implemented in the NBO program.^61^ The contribution of core electrons to the magnetic response was assessed using the removal of valence electron approximation.^53,54^ Ring-current strength susceptibilities (reported in nA/nT) were determined by numerically integrating the current density across a plane extending from the center of the ring to a region where the current density becomes negligible. The integration plane was placed 8 Bohr above and below the ring plane. Aromaticity was evaluated through the electron density of delocalized bonds (EDDB)^62^ using the EDDBRun scripts^63^ at the M06-2X/def2-QZVP level.

## Supplementary Material





## Notes

Interaction diagram for the [(C_2_H_4_)­PtCl_3_]^−^ anion and Cartesian coordinates of the [(C_2_H_4_)­PtCl_3_]^−^ anion at different levels, as well as those of the species involved in the isodesmic reactions (PDF)
An additional file containing all the data used to construct the *B* ^*ind*^_*y*_ profiles (XLSX)
