# Supplementary material for: Aromaticity in Ancient Zeise’s Salt
Source: Inorg Chem. 2025 Sep 29;64(40):20157–62. doi: 10.1021/acs.inorgchem.5c03091 (PMC12522126; doi:10.1021/acs.inorgchem.5c03091)
Supplement: Supplementary file 1 [file ic5c03091_si_001.pdf]

Supporting Information

Aromaticity in Ancient Zeise's Salt

Ankur K. Guha,[1,*] Amlan J. Kalita,[1,2] Mesías Orozco-Ic,[3] María A. Fernández-Herrera,[4] and Gabriel Merino.[4,*]

[1]Advanced Computational Chemistry Centre, Department of Chemistry, Cotton University, Panbazar, Guwahati, Assam, India-781001; ankurkantiguha@gmail.com

[2]Department of Chemistry, Nabajyoti College, Kalgachia, Assam, India-781319

[3]Instituto de Ciencias Físicas, Universidad Nacional Autónoma de México, 62210 Cuernavaca, México.

[4]Departamento de FísicaAplicada, Centro de Investigación y de Estudios Avanzados, Unidad Mérida. Km 6 Antigua Carretera a Progreso. Apdo. Postal 73, Cordemex, 97310, Mérida, Yuc., México; gmerino@cinvestav.mx

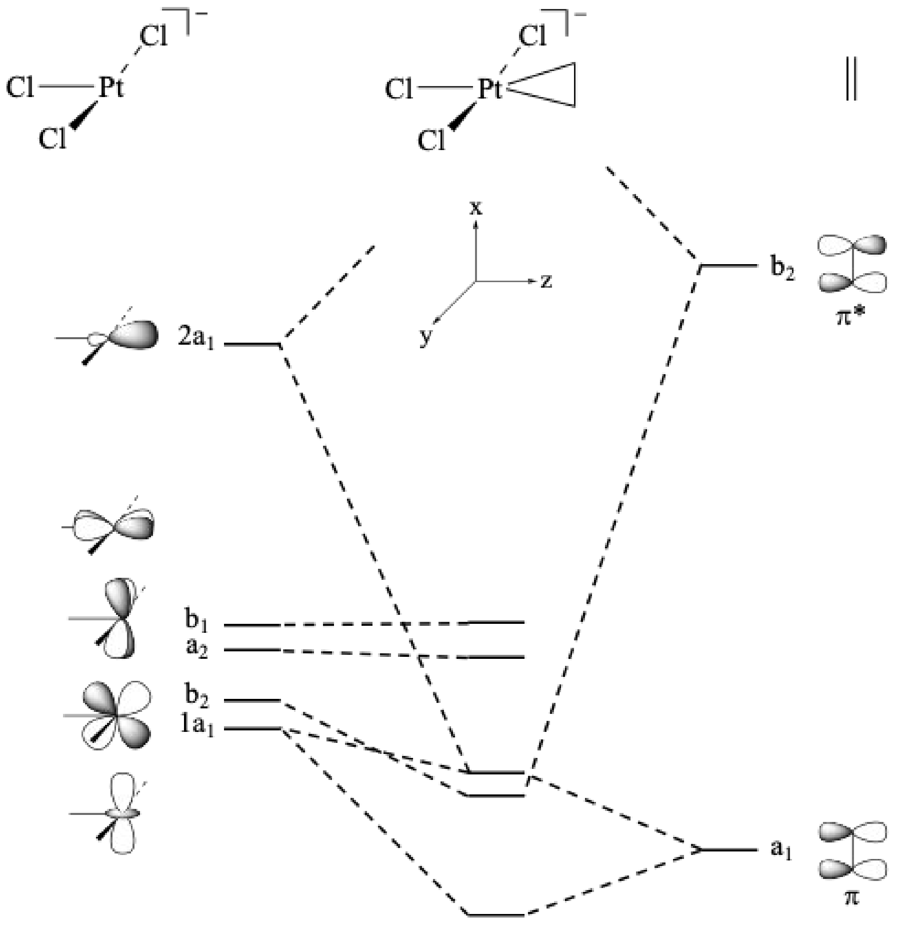

Figure S1. Interaction diagram for the $[(C_2H_4)PtCl_3]^-$ anion. Adapted from Ref. 14.

Cartesian Coordinates

$[PtCl_3(C_2H_4)]^-$

BHandHLYP/def2-QZVP

C	-0.689604000	0.000000000	2.090429000
H	-1.229877000	-0.914398000	2.239685000
H	-1.229877000	0.914398000	2.239685000
Pt	0.000000000	0.000000000	0.065439000
Cl	0.000000000	-2.333829000	0.112002000
Cl	0.000000000	2.333829000	0.112002000
Cl	0.000000000	0.000000000	-2.265296000
C	0.689604000	0.000000000	2.090429000
H	1.229877000	-0.914398000	2.239685000
H	1.229877000	0.914398000	2.239685000

CAM-B3LYP/def2-QZVP

C	0.000000000	0.695201000	2.044203000
H	-0.919795000	1.236220000	2.209639000
H	0.919795000	1.236220000	2.209639000
Pt	0.000000000	0.000000000	0.037122000
Cl	-2.330105000	0.000000000	0.076538000
Cl	2.330105000	0.000000000	0.076538000
Cl	0.000000000	0.000000000	-2.286284000
C	0.000000000	-0.695201000	2.044203000
H	-0.919795000	-1.236220000	2.209639000
H	0.919795000	-1.236220000	2.209639000

MN15/def2-QVZP

C	-0.697550000	0.000000000	2.068629000
H	-1.238049000	-0.923767000	2.228588000
H	-1.238049000	0.923767000	2.228588000
Pt	0.000000000	0.000000000	0.076350000
Cl	0.000000000	-2.307686000	0.128963000
Cl	0.000000000	2.307686000	0.128963000
Cl	0.000000000	0.000000000	-2.222145000
C	0.697550000	0.000000000	2.068629000
H	1.238049000	-0.923767000	2.228588000
H	1.238049000	0.923767000	2.228588000

mPW1PW91/def2-QZVP

C	-0.698110000	0.000000000	2.070054000
H	-1.238792000	-0.921665000	2.236359000
H	-1.238792000	0.921665000	2.236359000
Pt	0.000000000	0.000000000	0.078716000
Cl	0.000000000	-2.319469000	0.116968000
Cl	0.000000000	2.319469000	0.116968000
Cl	0.000000000	0.000000000	-2.234460000
C	0.698110000	0.000000000	2.070054000

H	1.238792000	-0.921665000	2.236359000
H	1.238792000	0.921665000	2.236359000

PBE0/def2-QZVP

C	0.000000000	0.699065000	2.023595000
H	-0.923365000	1.239748000	2.191995000
H	0.923365000	1.239748000	2.191995000
Pt	0.000000000	0.000000000	0.038333000
Cl	-2.315487000	0.000000000	0.076046000
Cl	2.315487000	0.000000000	0.076046000
Cl	0.000000000	0.000000000	-2.272156000
C	0.000000000	-0.699065000	2.023595000
H	-0.923365000	-1.239748000	2.191995000
H	0.923365000	-1.239748000	2.191995000

TPSS/def2-QZVP

C	-0.705835000	0.000000000	2.072140000
H	-1.247278000	-0.924975000	2.244079000
H	-1.247278000	0.924975000	2.244079000
Pt	0.000000000	0.000000000	0.072328000
Cl	0.000000000	-2.340234000	0.114299000
Cl	0.000000000	2.340234000	0.114299000
Cl	0.000000000	0.000000000	-2.257788000
C	0.705835000	0.000000000	2.072140000
H	1.247278000	-0.924975000	2.244079000
H	1.247278000	0.924975000	2.244079000

ωB97XD/def2-QZVP

C	-0.696713000	0.000000000	2.077028000
H	-1.237820000	-0.921120000	2.246061000
H	-1.237820000	0.921120000	2.246061000
Pt	0.000000000	0.000000000	0.069254000
Cl	0.000000000	-2.330439000	0.105107000
Cl	0.000000000	2.330439000	0.105107000
Cl	0.000000000	0.000000000	-2.254031000
C	0.696713000	0.000000000	2.077028000
H	1.237820000	-0.921120000	2.246061000
H	1.237820000	0.921120000	2.246061000

M06-2X/def2-QZVP

Sum of electronic and zero-point Energies= -1578.644669
Sum of electronic and thermal Energies= -1578.635993
Sum of electronic and thermal Enthalpies= -1578.635049
Sum of electronic and thermal Free Energies= -1578.679767

C	0.000000000	-0.698681000	2.043163000
H	0.921949000	-1.237160000	2.209107000
H	-0.921949000	-1.237160000	2.209107000
Pt	0.000000000	0.000000000	0.073683000

Cl	2.336410000	0.000000000	0.118819000
Cl	-2.336410000	0.000000000	0.118819000
Cl	0.000000000	0.000000000	-2.278679000
C	0.000000000	0.698681000	2.043163000
H	0.921949000	1.237160000	2.209107000
H	-0.921949000	1.237160000	2.209107000

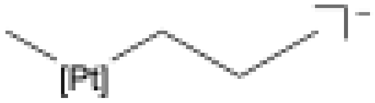

M06-2X/def2-QZVP

Sum of electronic and zero-point Energies=	*-1658.363882*
Sum of electronic and thermal Energies=	*-1658.350793*
Sum of electronic and thermal Enthalpies=	*-1658.349849*
Sum of electronic and thermal Free Energies=	*-1658.405805*

C	-0.974338000	-1.569241000	0.000000000
H	-1.591564000	-1.630637000	-0.893845000
H	-1.591564000	-1.630637000	0.893845000
Pt	-0.104656000	0.242592000	0.000000000
Cl	-0.142591000	0.292077000	2.326177000
Cl	-0.142591000	0.292077000	-2.326177000
Cl	2.368069000	0.198702000	0.000000000
C	-2.024391000	0.868006000	0.000000000
H	-1.978434000	1.957692000	0.000000000
H	-2.537034000	0.530797000	-0.896924000
H	-2.537034000	0.530797000	0.896924000
C	0.120435000	-2.615368000	0.000000000
H	0.755295000	-2.477825000	-0.874416000
H	0.755295000	-2.477825000	0.874416000
C	-0.490963000	-4.017010000	0.000000000
H	-1.114324000	-4.171412000	-0.881228000
H	-1.114324000	-4.171412000	0.881228000
H	0.285662000	-4.780988000	0.000000000

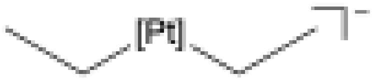

M06-2X/def2-QZVP

Sum of electronic and zero-point Energies=	*-1658.365925*
Sum of electronic and thermal Energies=	*-1658.352901*
Sum of electronic and thermal Enthalpies=	*-1658.351957*
Sum of electronic and thermal Free Energies=	*-1658.407561*

C	0.000000000	-1.302995000	1.559388000
H	-0.892441000	-1.129449000	2.155452000
H	0.892441000	-1.129449000	2.155452000
Pt	0.000000000	0.000000000	0.014678000

Cl	2.332412000	0.000000000	0.055593000
Cl	-2.332412000	0.000000000	0.055593000
Cl	0.000000000	0.000000000	-2.441516000
C	0.000000000	1.302995000	1.559388000
H	-0.892441000	1.129449000	2.155452000
H	0.892441000	1.129449000	2.155452000
C	0.000000000	-2.688106000	0.944651000
H	-0.884175000	-2.840487000	0.327356000
H	0.884175000	-2.840487000	0.327356000
H	0.000000000	-3.447590000	1.732170000
C	0.000000000	2.688106000	0.944651000
H	0.884175000	2.840487000	0.327356000
H	-0.884175000	2.840487000	0.327356000
H	0.000000000	3.447590000	1.732170000

CH_3CH_3

M06-2X/def2-QZVP

Sum of electronic and zero-point Energies= -79.739613
Sum of electronic and thermal Energies= -79.736127
Sum of electronic and thermal Enthalpies= -79.735183
Sum of electronic and thermal Free Energies= -79.761027

6	0.000000000	0.000000000	-0.761450000
1	0.000000000	1.014202000	-1.156717000
1	0.878325000	-0.507101000	-1.156717000
1	-0.878325000	-0.507101000	-1.156717000
6	0.000000000	0.000000000	0.761450000
1	-0.878325000	0.507101000	1.156717000
1	0.000000000	-1.014202000	1.156717000
1	0.878325000	0.507101000	1.156717000

C_3H_6

M06-2X/def2-QZVP

Sum of electronic and zero-point Energies= -117.808747
Sum of electronic and thermal Energies= -117.805398
Sum of electronic and thermal Enthalpies= -117.804454
Sum of electronic and thermal Free Energies= -117.831318

C	-0.864791000	0.000000000	0.000000000
C	0.432395000	0.748931000	0.000000000
C	0.432395000	-0.748931000	0.000000000
H	-1.447717000	0.000000000	0.907408000
H	-1.447717000	0.000000000	-0.907408000
H	0.723859000	1.253760000	0.907408000
H	0.723859000	1.253760000	-0.907408000
H	0.723859000	-1.253760000	-0.907408000
H	0.723859000	-1.253760000	0.907408000

C_5H_{12}

M06-2X/def2-QZVP

Sum of electronic and zero-point Energies=	-197.587429
Sum of electronic and thermal Energies=	-197.580378
Sum of electronic and thermal Enthalpies=	-197.579434
Sum of electronic and thermal Free Energies=	-197.617038

C	-1.272471000	0.000000000	-0.520397000
H	-1.269959000	-0.873511000	-1.175985000
C	-2.535096000	0.000000000	0.331244000
H	-2.568982000	-0.879396000	0.974223000
H	-2.568982000	0.879396000	0.974223000
H	-1.269959000	0.873511000	-1.175985000
C	2.535096000	0.000000000	0.331244000
H	2.568982000	0.879396000	0.974223000
H	2.568982000	-0.879396000	0.974223000
C	1.272471000	0.000000000	-0.520397000
H	1.269959000	0.873511000	-1.175985000
H	1.269959000	-0.873511000	-1.175985000
H	3.434550000	0.000000000	-0.281716000
C	0.000000000	0.000000000	0.316437000
H	0.000000000	-0.874084000	0.974151000
H	0.000000000	0.874084000	0.974151000
H	-3.434550000	0.000000000	-0.281716000
